# Evaluation of EGFR and COX pathway inhibition in human colon organoids of serrated polyposis and other hereditary cancer syndromes

**DOI:** 10.1007/s10689-024-00370-7

**Published:** 2024-04-12

**Authors:** Priyanka Kanth, Mark W. Hazel, John C. Schell, Jared Rutter, Ruoxin Yao, Alyssa P. Mills, Don A. Delker

**Affiliations:** 1 MedStar Georgetown University Hospital, Washington, DC 20007 USA; 2 Division of Gastroenterology, Department of Internal Medicine, University of Utah, Salt Lake City, UT 84132 USA; 3 Department of Biochemistry, University of Utah, Salt Lake City, UT 84132 USA; 4 Integrative Bioinformatics, National Institutes of Environmental Health Sciences, 111 TW Alexander Drive, Research Triangle Park, NC 27709 USA

**Keywords:** Colon organoid, Colon polyp, Erlotinib, Familial adenomatous polyposis, Gene expression, Lynch syndrome, Serrated polyposis, Sulindac

## Abstract

Serrated polyposis syndrome (SPS) presents with multiple sessile serrated lesions (SSL) in the large intestine and confers increased colorectal cancer (CRC) risk. However, the etiology of SPS is not known. SSL-derived organoids have not been previously studied but may help provide insights into SPS pathogenesis and identify novel biomarkers and chemopreventive strategies. This study examined effects of EGFR and COX pathway inhibition in organoid cultures derived from uninvolved colon and polyps of SPS patients. We also compared with organoids representing the hereditary gastrointestinal syndromes, Familial Adenomatous Polyposis (FAP) and Lynch syndrome (LS). Eighteen total organoid colon cultures were generated from uninvolved colon and polyps in SPS, FAP, LS, and non-syndromic screening colonoscopy patients. *BRAF* and *KRAS* mutation status was determined for each culture. Erlotinib (EGFR inhibitor) and sulindac (COX inhibitor) were applied individually and in combination. A 44-target gene custom mRNA panel (including WNT and COX pathway genes) and a 798-gene microRNA gene panel were used to quantitate organoid RNA expression by NanoString analysis. Erlotinib treatment significantly decreased levels of mRNAs associated with WNT and MAPK kinase signaling in organoids from uninvolved colon from all four patient categories and from all SSL and adenomatous polyps. Sulindac did not change the mRNA profile in any culture. Our findings suggest that EGFR inhibitors may contribute to the chemopreventive treatment of SSLs. These findings may also facilitate clinical trial design using these agents in SPS patients. Differentially expressed genes identified in our study (*MYC, FOSL1*, *EGR1, IL33, LGR5 and FOXQ1*) may be used to identify other new molecular targets for chemoprevention of SSLs.

**Supplementary Information:**

The online version contains supplementary material available at 10.1007/s10689-024-00370-7.

## Introduction

Colon cancer is the second leading cause of cancer-related deaths in the United States and third most common cancer in men and women [1]. Hereditary gastrointestinal cancer syndromes such as serrated polyposis syndrome (SPS), familial adenomatous polyposis (FAP) and Lynch syndrome (LS) carry a high risk for development of colorectal cancer (CRC). These high-risk conditions require close surveillance with regular colonoscopies for prevention and management of colorectal cancer [2]. A few chemopreventive agents have been studied in FAP and Lynch cohorts to prevent colon polyps and CRC. However, such studies have been limited in their understanding of drug effects on colon stem cell proliferation, differentiation, and phenotype. The effect of chemopreventive agents in SPS remains unexplored.

Multiple studies suggest that the epidermal growth factor receptor (EGFR) pathway plays a role in colon cancer development. Increased EGFR expression has been shown in adenomas and colon cancer in human and animal studies [3, 4]. EGFR signaling is needed for adenoma growth, and EGFR inhibitors cause regression of intestinal adenomas [5–8]. A prior clinical study, however, did not show a statistical reduction in biomarkers of EGFR signaling in aberrant crypt foci using multiple doses of erlotinib (an EGFR inhibitor) over a short-term duration [9]. Sulindac, a nonsteroidal anti-inflammatory drug (NSAID) and a prostaglandin-endoperoxide synthase (COX) pathway inhibitor, has been studied in the prevention of colon polyp formation in patients with FAP [10–12]. The underlying molecular mechanism of its action on FAP adenomas is, however, not well understood. A recent phase II clinical trial of a combination of erlotinib and sulindac showed promising effects on duodenal and colorectal polyp burden regression in patients with FAP [13, 14]. The roles of EGFR or COX pathways have been less studied in Lynch syndrome. NSAIDs have shown promising results in prevention of CRC in Lynch syndrome [15]. Aspirin, a COX pathway inhibitor, has been studied in clinical trials for chemoprevention of Lynch syndrome and sporadic CRC [15, 16]. EGFR and COX pathway inhibitors have not been studied in the prevention of sessile serrated lesions (SSLs) or in serrated polyposis syndrome. These agents have not been explored in a colon organoid model, although doing so can provide interesting insights into the activity of these inhibitors at the cellular level.

Organoid cultures provide an excellent translational model to study the mechanisms underlying human diseases, including cancer, that develop from abnormal epithelial stem cell growth and differentiation [17]. Human colon organoids can be derived from normal colon, colonic polyps or cancer tissues to study the effects of candidate drugs in many disease conditions [18, 19]. Previous molecular studies show increased epidermal growth factor receptor (EGFR) and COX signaling in colon polyps [20–22]. Our previous gene expression analyses in serrated polyps showed increased expression of *PTGS2* (COX2) mRNA in sessile serrated lesions (previously termed “sessile serrated adenoma/polyps”) [23]. The purpose of this study was three-fold: first, develop organoid models of uninvolved colon and colon polyps from subjects with SPS; second, compare EGFR and COX signaling in colon organoids derived from SPS, FAP and Lynch syndrome; and third, determine the effects of EGFR (erlotinib) and COX (sulindac) inhibitors on colon organoid stem cell growth and differentiation in the three high-risk groups. Our study indicates that colon organoids can be derived from high-risk populations and can provide valuable insights into the molecular and cellular nature of organoids derived from colon polyps. We further show that erlotinib significantly reduces organoid stem cell growth independent of patient cohort or underlying mutational status while little if any effect is observed with sulindac. These studies provide important data to support the use of EGFR inhibitors as a therapeutic strategy for SPS patients.

## Materials and methods

### Sample collection

Freshly obtained colon tissue biopsies were collected from patients with AFAP (*n* = 2), Lynch (*n* = 2), SPS (*n* = 4) and normal screening colonoscopy patients (*n* = 2) seen at the University of Utah Healthcare Hospitals and Huntsman Cancer Hospital. Table 1 describes patient demographics. Colon samples were obtained from ascending and sigmoid colon. A total of 18 colon organoid cultures were created from these 10 patients (Table 2). Ten organoid cultures were created from SSLs (*n* = *5*) and macroscopically uninvolved tissue (*n* = *5*) from 4 SPS patients. Four organoid cultures were created from two adenomatous polyps and two uninvolved tissue samples from 2 AFAP patients. Four additional organoid cultures were created from two Lynch and two control patients (macroscopically uninvolved tissue only, Table 2). The study was approved by the Institutional review board. Colon organoids were isolated using the protocol developed by Sato and colleagues [18, 24].

**Table 1:** Patient demographics

Pt No	Diagnosis	Age	Gender	Ethnicity	Personal history of cancer	Family history of Colon cancer	Smoking	BMI	Aspirin usage
1	Control	67	M	Native American	Breast	No	Former	28	Yes
2	Control	54	M	Caucasian	No	No	Never	55	No
3	LS	39	F	Caucasian	No	No	Never	53	No
4	LS	38	M	Caucasian	No	Yes	Current	26	No
5	SPS	69	M	Caucasian	No	No	Former	21	No
6	SPS	40	F	Caucasian	No	No	Never	23	No
7	SPS	40	F	Caucasian	No	No	Former	39	No
8	SPS	70	F	Caucasian	No	No	Former	NA	Yes
9	AFAP	62	F	Caucasian	No	Yes	Former	27	No
10	AFAP	72	M	Caucasian	No	No	Former	42	No

*LS* Lynch syndrome, *SPS* serrated polyposis syndrome, *AFAP* attenuated familial adenomatous polyposis, *NA* not available

**Table 2:** 18 Organoid cultures used for mRNA and/or miRNA NanoString analysis

								NanoString Analyses	
Cohort	Patient	Tissue	Culture ID	Location	BRAF V600	KRAS codons 12–13	Passage	mRNA	miRNA
CTRL	1	Normal	354C-SC	Sigmoid	wt	wt	4	x	
CTRL	2	Normal	355C-SC	Sigmoid	wt	wt	3	x	
AFAP	9	Normal	353F-SC	Sigmoid	wt	wt	4	x	
AFAP	10	Normal	359F-AC	Ascending	wt	wt	8	x	x
AFAP	10	AP	359F-CP	Cecum	wt	wt	17	x	x
AFAP	10	AP	359F-TP	Transverse	mut	wt	8	x	x
LS	3	Normal	352L-SC	Sigmoid	wt	wt	4	x	
LS	4	Normal	356L-SC	Sigmoid	wt	wt	3	x	
SPS	7	Normal	342S-AC	Ascending	wt	wt	4	x	x
SPS	7	Normal	342S-SC	Sigmoid	wt	wt	4		x
SPS	7	SSL	342S-HFP	Hepatic Flex	wt	wt	4	x	x
SPS	7	SSL	342S-TP	Transverse	wt	wt	4	x	x
SPS	5	Normal	357S-SC	Sigmoid	wt	wt	11	x	
SPS	5	SSL	357S-TP	Transverse	mut	wt	11	x	x
SPS	6	Normal	358S-AC	Ascending	wt	wt	5	x	x
SPS	6	SSL	358S-AP	Ascending	wt	wt	4	x	x
SPS	8	Normal	360S-AC	Ascending	wt	wt	3	x	x
SPS	8	SSL	360S-AP	Ascending	mut	wt	8	x	x

### Organoid cultures

At each passage, human colon organoids were rinsed in fresh basal culture medium, spun down (500 rcf, 5′ 4 °C), fragmented by moving in and out of a pipet tip, and plated within new 7 μl spots of 42% MatriGel Matrix (Corning 356,231) plus 58% 1× growth media. Our standard growth media, containing recombinant murine Wnt3A, EGF and Noggin, 5% mRSPO1-Fc-conditioned medium from 293 T cells, and GSK-3 inhibitor CHIR 99021, was modified slightly from that of our earlier report [25]. This study’s growth media was the same except for these changes: (a) 50 mg Primocin (InvivoGen) was included in each 500 mls of basal cell medium (i.e., the Advanced DMEM/F12, HEPES, Glutamax and Penicillin–Streptomycin); (b) valproic acid and UI-5099 were omitted; (c) 10 nM recombinant human (LEU-15]-Gastrin I (Sigma G9145) was included; (d) 10 mM Nicotinamide (Sigma N0636), 500 nM A83-01 (R & D Systems 2939), and 10 μM SB202190 (Sigma S7067) were included; (e) 10uM Y-27632 dihydrochloride (R & D Systems 1254) was included, but only in the media initially added to the MatriGel spots and initial each well’s initial media volume (but not after Day 3).

Cultures were grown in standard, TC-treated tissue culture plates (Genesee Scientific, #25–105): one 7 μl spot with 175 μl media per well in 48-well tissue culture plates (for erlotinib/sulindac treatments), five 7 μl spots with 500ul media per well in a 24-well plate (for antibody-staining experiments) or twenty 7 μl spots with 2 ml media per well in a 6-well plate (for general culture). Peripheral wells in 48- and 24-well plates held PBS and were not used for cultures due to their increased evaporation. Media was routinely fully changed first at Day 3 post passage and every two days thereafter. Passaging routinely was done at Day 7 or 8. All results here are based on cultures at passages between 3 and 15. Organoid cultures were viewed daily for growth and morphology using a Zeiss Invertoskop (inverted phase contrast microscope). Brightfield and fluorescent images of each organoid culture were taken using a Thermo Fisher Scientific EVOS auto color microscope (Supplemental Methods, https://figshare.com/s/e4cf0874fbbe8798942c).

### Drug treatments

Inhibition of COX and EGFR pathways in colon organoids were performed with commercially available small chemicals sulindac sulfide (Sigma-Aldrich S3131) and erlotinib hydrochloride (Selleck Chemicals S1023). The drug concentrations (0.5 and 2 μM for erlotinib; 20 and 100 μM for sulindac) used were determined from previously published cell culture experiments and the predicted blood concentration needed to show efficacy in clinical studies [26–28]. Because 0.5 and 2 μM erlotinib showed similar effects on organoid gene expression (data not shown), 0.5 μM erlotinib was used throughout this study. Erlotinib and sulindac effects on gene expression were evaluated at 6 and 24 h. Drugs were studied individually and in combination to understand their synergistic effects on gene expression of different signaling pathways. A complete list of cultures used for drug treatments are shown in Table S4.

### Embedding and preparations of sections

Two 6-well-plate tissue culture wells (3.5 cm diameter) that contained a total of ~ 45 7ul MatriGel spots of Day 6 or 7 (post-passage) organoid cultures were used to generate each FFPE block. MatriGel spots containing the organoids were gently scraped, fixed in 10% Buffered Formalin Phosphate (Fisher Scientific) for ~ 20 h at room temperature, and shifted into 70% EtOH. Fixed organoids were spun 400 g 5 min, and 200ul containing them was combined with 350ul warmed HistoGel (Thermo Scientific, #HG-4000-012). Following gentle, brief mixing by pipetting, each prep’s 550ul was transferred to a chilled 10 × 10 × 5 mm plastic cryomold until solidification and then placed in 70% EtOH until routine processing into a FFPE block by the Biorepository and Molecular Pathology Shared Resource at the Huntsman Cancer Institute, University of Utah. 4 μm sections were stained with hematoxylin and eosin, or stained with 1%, pH 2.5 Alcian Blue (VWR, #1003A) for 30 min and then Nuclear Fast Red (Newcomer Supply, #604451) for 5 min. Brightfield images were taken using Thermo Fisher Scientific EVOS auto color microscope.

### RNA extraction from organoids

Total RNA was isolated from 17 individual organoid cultures using the Zymo Research Direct-zol protocol. A minimum of 100 organoids (10^5^–10^6^ cells) from each treatment group was homogenized by gentile shaking in 1 ml of TRIzol RNA isolation reagent. The cell homogenate was passed through a Zymo-Spin column and the retained RNA washed several times with alcohol and finally eluted with nuclease free water.

### RNA expression analysis using NanoString

A gene panel of 44 target and 4 housekeeping genes was constructed to examine RNA expression differences related to organoid culture phenotype and drug treatment (Table S1). In all, 128 organoid samples were used for NanoString analysis. Our gene panel included genes specific to the WNT signaling, EGFR and COX pathways and cell marker specific genes. Ten genes targeting EGFR and COX pathway were selected from our prior publication [22]. These genes include *CXCL5, PTGS2, EGFR, UCP2, CCL5, FST, HPGD, FOSL1, ERBB4* and *EGR1*. Seven genes (*TRNP1*, *CRYBA2, ZIC2, ZIC5, MUC6, SEMG1* and *FSCN1)* specific for sessile serrated lesions (SSLs) will be included from our prior study in the gene panel to study likely markers of serrated pathway [23]. Gene markers of cancer field effect (*TET3, CLDN8, WFDC2* and *ZDHHC20*) and long non-coding RNAs known to be associated with colon cancer and from our recent study (*CASC19, FEZF1-AS1, CCAT1* and *CRNDE*) were also included in the NanoString panel. For microRNA analysis, a commercially available NanoString miRNA panel consisting of 798 miRNAs was used.

100 ng of total RNA were submitted to the Molecular Diagnostics core at the Huntsman Cancer Institute, University of Utah for quality control analysis and hybridization to a pooled set of custom probes complimentary to all genes in the 48 gene panel. The number of transcripts from each gene was counted using an nCounter Analysis System that identifies molecular barcodes to detect hundreds of unique transcripts in a single reaction. nSolver analysis software was used for data analysis with normalization of raw counts using positive control and housekeeping genes. Differentially expressed genes were determined using nSolver univariate or DESeq2 multivariate statistical analysis. Machine learning algorithms including hierarchical clustering and principal component analysis was performed using Cluster 3.0 to identify expression patterns and Java Treeview was used for developing heatmaps.

### *BRAF* and *KRAS* mutation analysis

Mutations in codon 600 of *BRAF* and codons 12 and 13 of *KRAS* were determined by Sanger sequencing using PCR primers as previously described [29].

## Results

### Organoid morphology and *BRAF/KRAS* mutation status

**Fig. 1:**
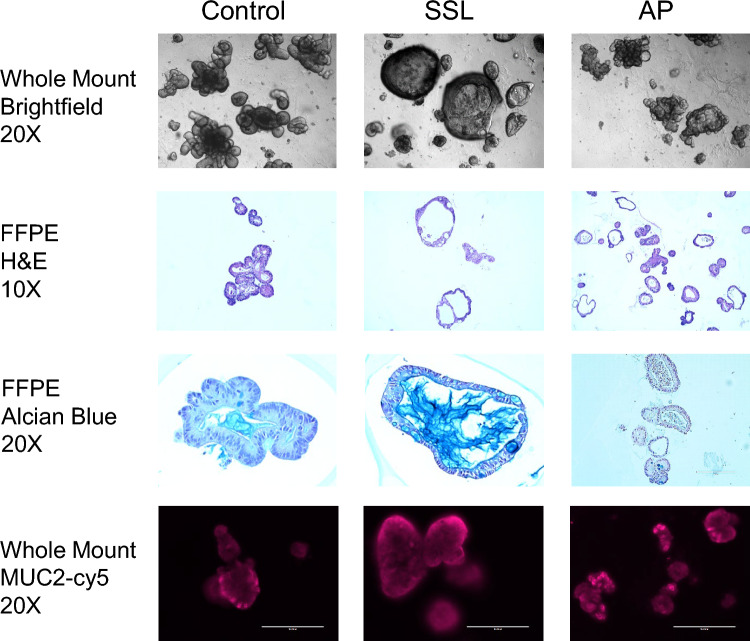
Photomicrographs of colon organoids derived from patients with Serrated Polyposis Syndrome (SPS) and Familial Adenomatous Polyposis (FAP). **Left**, colon organoids derived from uninvolved colon from an SPS patient by whole mount light microscopy (20× , top row), hematoxylin and eosin-stained tissue slide (10× , 2nd row), alcian blue stained tissue slide (3rd row, 20×) and fluorescence microscopy of mucin 2 (20× , bottom row) **Center**, colon organoids from a sessile serrated lesion (SSL). **Right**, colon organoids from an adenomatous polyp (AP) from FAP patient

Organoids were derived from freshly collected colon biopsies taken during routine screening colonoscopy. Colon organoids developed from macroscopically normal appearing mucosa showed similar morphology and growth patterns independent of patient cohort (Fig. S1). In contrast, organoids derived from colon polyps (SSLs from SPS patients and APs from AFAP patients) often showed different cell morphology and differentiation patterns. Figure 1 depicts organoids from ascending uninvolved colon and an SSL from a patient with SPS and an AP from a patient with FAP. Organoids derived from SSL and APs were more often spheroid in shape with SSLs containing *BRAF* V600E mutations showing the most severe spheroid phenotype (Fig. 1, Table 2, Table S4). Also, *BRAF* mutant SSLs showed decreased bud formation suggesting reduced cell differentiation (Fig. S2, Table S4). H&E stains of SSL and AP cultures also showed more dilated crypt domains when compared to uninvolved colon (Fig. 1). Colon organoids derived from APs from FAP patients showed less mucin secretion by Alcian blue staining compared to uninvolved colon and SSLs from SPS patients. Mucin 2 protein expression (goblet cell marker) by fluorescence microscopy showed changes in both the levels of protein and its cellular location in *BRAF* mutant organoids (Fig. 1, Fig. S2, Table S4). None of the organoid cultures showed mutations in codon 12 or 13 of *KRAS* (Table 2).

### NanoString analysis

#### RNA expression by patient cohort

**Fig. 2:**
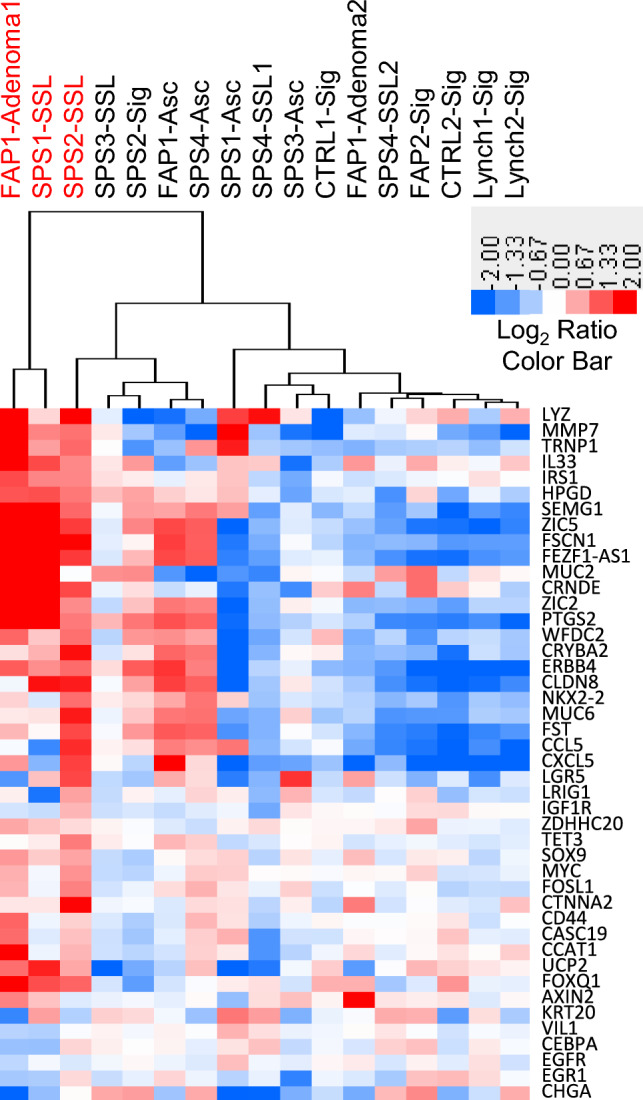
Hierarchical clustering of colon organoid RNA expression using a custom 48 gene NanoString panel. The log2 ratios for organoids derived from polyps (SSLs and APs) were determined by comparing each polyp to its paired uninvolved control. The log2 ratio for each uninvolved colon sample (CTRL, SPS, FAP and Lynch) was determined by comparing each uninvolved colon samples to the mean of all uninvolved colon samples. Red denotes increased expression, blue reduced expression, and white no change in expression. Organoid samples with red labeled text were positive for *BRAF* V600E mutation

Comparing expression of our 48-gene panel across uninvolved colon, SSLs, and APs, we identified significant differences in RNA expression dependent on *BRAF* V600E mutation status (Fig. 2, Table S2). Interestingly, 14 genes showed the highest expression based on *BRAF* V600E mutation in two SSLs and one AP. Overexpression of WNT (*MMP7*) and COX (*PTGS2*, *HPGD*) signaling genes were noted in these polyps. Five of seven genes specific for SSLs (*TRNP1, SEMG1, ZIC5*, *FSCN1* and *ZIC2*) were overexpressed in these polyps [23]. Two long non-coding RNAs, forebrain embryonic zinc finger 1 antisense 1 (*FEZF1-AS1*) and colorectal neoplasia differentially expressed (*CRNDE*), were overexpressed. Two genes previously associated with PI3K signaling, interleukin 33 (*IL33*) and insulin receptor substrate 1 (*IRS1*) and two cell marker genes, mucin 2 (*MUC2*) and lysozyme (*LYZ*), were also overexpressed in *BRAF* mutant organoids.

Comparing organoids derived from uninvolved colon across the four patient cohorts (CTRL, LS, FAP, SPS) we identified four genes that were differentially expressed by multivariate analysis, *FOXQ1, IGF1R, MMP7* and *TET3* (Fig. S3). *FOXQ1* showed lower expression across all three high risk cohorts (LS, FAP, SPS) compared to control patients. Uninvolved colon organoids from SPS patients showed lower expression of *IGF1R* and *TET3* compared to the other three patient cohorts. MMP7 was more highly expressed in uninvolved colon organoids from SPS patients compared to control and Lynch patients.

**Fig. 3:**
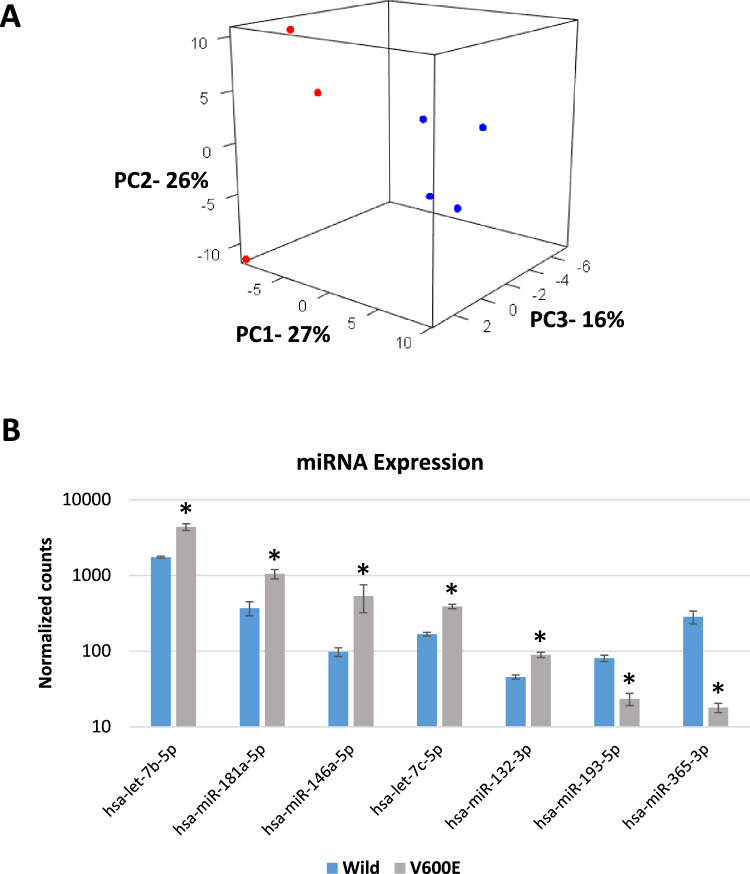
MicroRNA expression in colon organoids (**A**) Principal component analysis of microRNA expression in organoids derived from four SSLs from SPS patients and three APs from FAP patients. 3D figure shows first three components (PC1-PC3) accounting for approximately 70% of the variation in the data. Log2 ratios were calculated by comparing each polyp to its uninvolved control. Red denotes two SSLs and one AP with *BRAF* V600E mutation (*n* = 3) and blue denotes three SSLs and one AP without *BRAF* mutation (*n* = 4). (**B**) Relative expression of seven microRNAs (miRNAs) differentially expressed in three *BRAF* V600E mutant, two SSLs and one AP, compared to four *BRAF* wildtype, three SSLs and one AP, organoids. Bar graphs show the mean and standard error of normalized read counts for each miRNA. Statistical significance determined by DESeq2, FDR < 0.05

Comparing microRNA expression across SPS and FAP patient samples we saw a similar pattern of expression associated with *BRAF* mutation (Fig. 3A). Among the miRNAs most differentially expressed in *BRAF* mutant organoids were *MIR365A, MIR146A, LET7B, LET7C, MIR193A, MIR132* and *MIR181A* (Fig. 3B, Table S3).

#### RNA expression by drug treatment

**Fig. 4:**
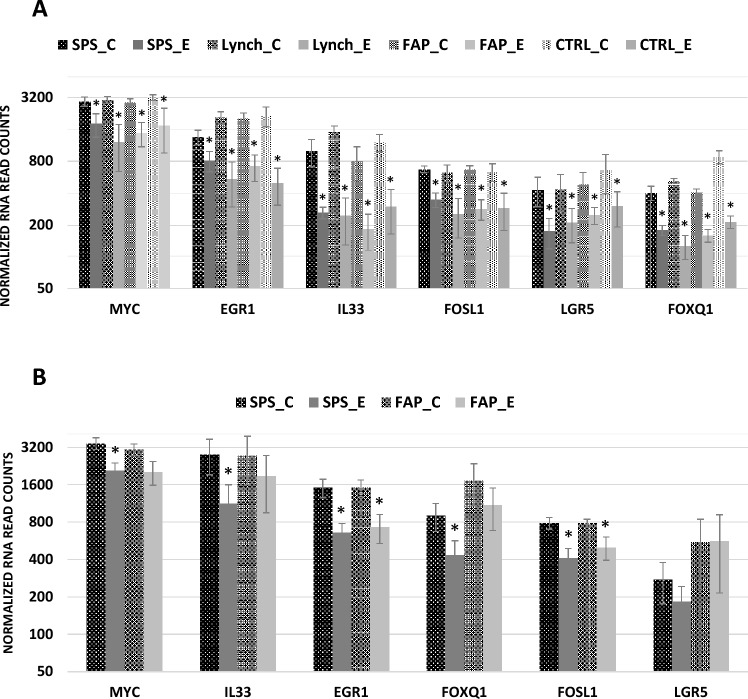
RNA expression in control and erlotinib treated colon organoids (**A**) RNA expression of *MYC, EGR1, IL33, FOSL1, LGR5* and *FOXQ1* in control (C) and erlotinib (E) treated organoids derived from uninvolved colon from SPS, FAP, Lynch and control (non-syndromic patients with average cancer risk) patient cohorts. Bar graphs show the mean and standard error (*n* = 4–6) of normalized read counts for each gene. Statistical significance determined by DESeq2, FDR < 0.05. (**B**) RNA expression of *MYC, EGR1, IL33, FOSL1, LGR5* and *FOXQ1* in control (C) and erlotinib (E) treated organoids derived from SSLs from SPS patients and APs from FAP patients. Bar graphs show the mean and standard error (*n* = 4–6) of normalized read counts for each gene. Statistical significance determined by DESeq2, FDR < 0.05

Erlotinib treatment resulted in significant decreases in target mRNA expression associated with WNT and MAPK kinase signaling in organoids derived from uninvolved colon from all patient cohorts (Fig. 4A). Among the mRNAs most differentially expressed were *MYC, FOSL1*, *EGR1, IL33, LGR5 and FOXQ1* (Fold −1.5 to −3.8, FDR < 0.01). These changes in mRNA expression were observed at both 6 and 24 h post exposure. Similar decreases in RNA expression were observed in organoids derived from SSLs and APs (Fig. 4B). Changes in the baseline expression of these mRNAs, except *FOXQ1*, was not different between patient cohorts. No changes in organoid morphology or growth were observed after 24 h of erlotinib treatment. However, decreased organoid growth was observed after 72 h and widespread cell death observed after 4 days (data not shown).

**Fig. 5:**
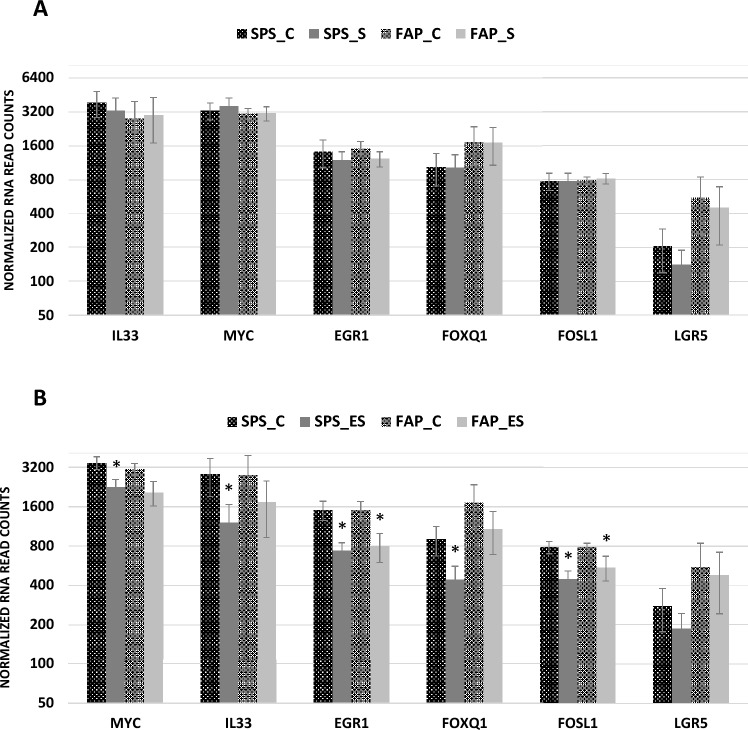
RNA expression in sulindac and erlotinib treated colon organoids (**A**) RNA expression of *MYC, EGR1, IL33, FOSL1, LGR5* and *FOXQ1* in control (C) and sulindac (S) treated organoids derived from uninvolved colon from SPS, FAP, Lynch and control patient cohorts. Bar graphs show the mean and standard error (*n* = 4–6) of normalized read counts for each gene. (**B**) RNA expression of *MYC, EGR1, IL33, FOSL1, LGR5* and *FOXQ1* in control (C) and erlotinib + sulindac combination (ES) treated organoids derived from SSLs from SPS patients and APs from FAP patients. Bar graphs show the mean and standard error (*n* = 4–6) of normalized read counts for each gene. Statistical significance determined by DESeq2, FDR < 0.05

Sulindac treatment alone did not significantly change mRNA expression at any time point in organoids derived from uninvolved colon, SSLs or APs (Fig. 5A). However, the combination of sulindac and erlotinib decreased the expression of *MYC, FOSL1*, *EGR1, IL33, LGR5 and FOXQ1* like erlotinib alone (Fig. 5B).

## Discussion

Human colon organoids provide a valuable model to study intestinal diseases including the opportunity to study the efficacy of chemopreventive agents. Even though hereditary colon cancer syndromes including FAP and LS generate a small proportion of all colon cancers, patients with these syndromes carry 40–100% risk of colon cancer development [2]. Chemoprevention of cancers in these syndromes is an area of much interest, with clinical trials in FAP attempting to explore colon and duodenal polyp reduction using drug treatments [13, 14]. Erlotinib (orally active EGFR tyrosine kinase inhibitor) and sulindac (COX inhibitor) were studied in a recent clinical trial that showed substantial polyp regression in duodenum and colon [13]. Erlotinib is a newer agent being studied for this condition and the initial trial showed promising results in FAP patients. On the other hand, sulindac has been extensively studied in the chemoprevention of FAP with moderate results [30].

The serrated pathway contributes up to 30% of all colon cancer and originates from sessile serrated lesions (SSL). Serrated polyposis syndrome (SPS) is an underdiagnosed condition with multiple SSLs and carries a high risk of colon cancer development. The serrated pathway has not been explored before by using human organoids from SPS patients. Recent studies attempt to describe the complexity of the serrated pathway in organoid models [21, 31, 32]. However, no chemopreventive studies have been done in patients with SPS. Our group has previously described differential gene expression in SSLs in patients with SPS and sporadic SSLs [23, 29, 33]. It is unclear if these gene markers could play a role in EGFR and COX pathways to colon cancer. In this study we show that organoids can be derived from patients with colon cancer syndromes including patients with SPS. In addition, organoids can be derived from SSLs and permit the testing of known and novel chemopreventive agents, like erlotinib and sulindac, which may reduce the progression of these lesions.

Many genes were differentially expressed in *BRAF* mutated (V600E) polyp organoids from FAP (AP) and SPS (SSL) patients when compared to *BRAF* wild-type organoids. Overexpression of WNT (*MMP7, CD44*), COX (*PTGS2*, *HPGD*) and MAP kinase (*FOSL1*, *EGR1*) signaling genes was noted in *BRAF* mutant organoids. Five of the seven genes specific for SSLs (*TRNP1, SEMG1, ZIC5*, *FSCN1* and *ZIC2*) were also overexpressed in these organoids. These five genes are part of a seven gene panel developed by our laboratory to differentiate between benign and precancerous serrated lesions [29].

Erlotinib treatment showed marked differences in organoid gene expression across all patient cohorts and sample types. Among the genes most changed by treatment were *MYC, FOSL1*, *EGR1, IL33, LGR5 and FOXQ1. MYC* is an oncogene and encourages progression to neoplasia in many malignancies including colorectal cancer [34, 35]. Also, mutation of the *APC* gene has been shown to activate c-*MYC* [36]. *EGR1* is a downstream *MYC* target gene with a role in cell apoptosis [35]. Upregulation of *EGR1* has been demonstrated by NSAIDs (such as sulindac and celecoxib) in animal model and human cell line studies [37, 38]. *FOSL1,* also known as *FRA-1,* belongs to the FOS gene family and forms the transcription factor complex AP-1. The genes in this complex are targets of beta-catenin signaling pathway and promote colon cancer progression as shown in human colon cancer cell lines [39].

PI3K pathway mutations are also found in cancers that have arisen from the serrated neoplasia pathway [40]. Two PI3K signaling genes (*IL33* and *IRS1*) were overexpressed in organoids from *BRAF* mutated SSLs and AP. IL33 acts as a tumor suppressor gene by inhibiting the development of colon cancer in sporadic and colitis animal models [41, 42]. *FOXQ1* is another PI3K signaling and WNT target gene, and its overexpression of is associated with colon tumor formation and metastasis [43–45]. *LGR5* is a stem cell marker, plays a role in the WNT-signaling pathway, and its overexpression has been shown in CRC tissues [46, 47]. Interestingly, a recent study showed *LGR5*-negative cells can drive metastatic colorectal cancer [48].

*IRS1*, an insulin signaling pathway gene, expressed stronger immunostaining in colon adenomas from FAP and in colorectal cancers and is suggested to play a role in colon tumor development [49, 50]. *MUC2* and *LYZ* are cell-type specific markers for goblet and Paneth cells, respectively. Two long non-coding RNAs, forebrain embryonic zinc finger 1 antisense 1 (*FEZF1-AS1*) and colorectal neoplasia differentially expressed (*CRNDE*), were overexpressed and have been previously associated with colon cancer. *FEZF1-AS1* expression has been associated with poor survival and tumor metastasis in colon cancer, and it activates STAT3 signaling [51]. *CRNDE* is highly expressed in colorectal adenomas and colon cancer [52] and may play a role through PI3K signaling in other cancers [53].

A number of microRNAs showed significant differential expression in colon polyps. *MIR365a-3p, MIR146a-5p* and *MIR193a-5p* showed up to 20-fold change between uninvolved colon and *BRAF* mutant SSLs and AP. *MIR365* has been shown to be down regulated in colon cancer tissue and may suggest poor prognosis in patients with colon cancer [54]. *MIR146* has a role in initiation of colon cancer through stem cell alterations [55].

Sulindac treatment did not show changes in organoid gene expression like erlotinib. This may be due to the lack of immune and submucosal cells in our organoid cultures. Such non-epithelial cells may be necessary to mediate the immunological effects of sulindac. Understanding these chemotherapeutic agents at the molecular level in SSLs may provide further insight into effects of these drugs in target pathways to colon cancer.

In conclusion, we present data that describe a novel colon organoid model of serrated polyposis for testing the therapeutic potential of erlotinib and other candidate therapeutics for the treatment of colon SSLs. We show that *BRAF* V600E mutations greatly influence both organoid morphology and gene expression. Our findings also support the therapeutic potential of erlotinib in inhibiting SSL growth in patients with SPS. In contrast, sulindac did not show a similar effect and may be due to the absence of immune cells in our organoid model.

## Supplementary Information

Below is the link to the electronic supplementary material.

Supplementary file1 (PDF 117 KB)

Supplementary file2 (PDF 129 KB)

Supplementary file3 (PDF 70 KB)

Supplementary file4 (XLS 37 KB)

Supplementary file5 (XLS 40 KB)

Supplementary file6 (XLS 132 KB)

Supplementary file7 (XLSX 12 KB)

Supplementary file8 (DOCX 15 KB)
