# Supplementary figures and images for: Evaluation of EGFR and COX pathway inhibition in human colon organoids of serrated polyposis and other hereditary cancer syndromes
Source: Fam Cancer. 2024 Apr 12;23(4):479–89. doi: 10.1007/s10689-024-00370-7 (PMC11512843; doi:10.1007/s10689-024-00370-7)

## Supplemental Figure 1

FAP uninvolved

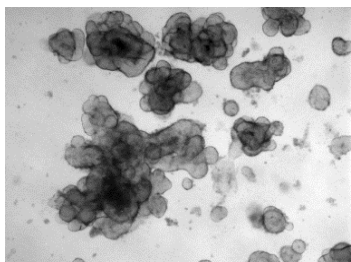

Lynch uninvolved

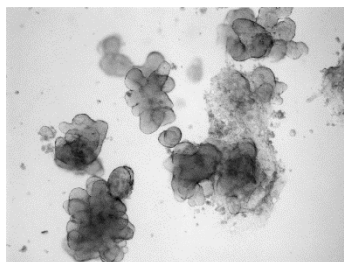

Supplement: Supplementary file 1 — Supplementary file1 (PDF 117 KB) [file 10689_2024_370_MOESM1_ESM.pdf]

Supplemental Figure 2

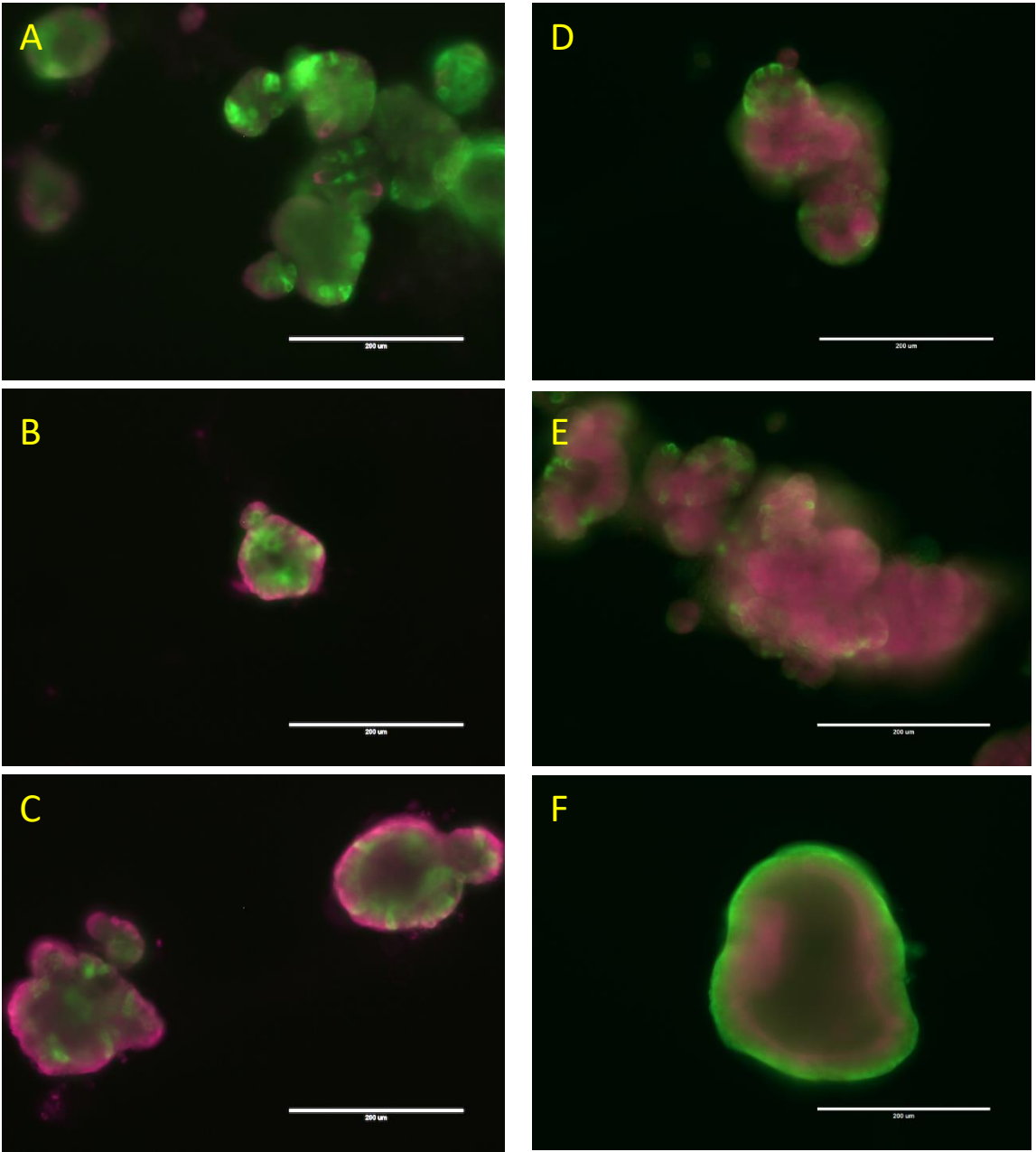

Supplement: Supplementary file 2 — Supplementary file2 (PDF 129 KB) [file 10689_2024_370_MOESM2_ESM.pdf]

# Supplemental Figure 3

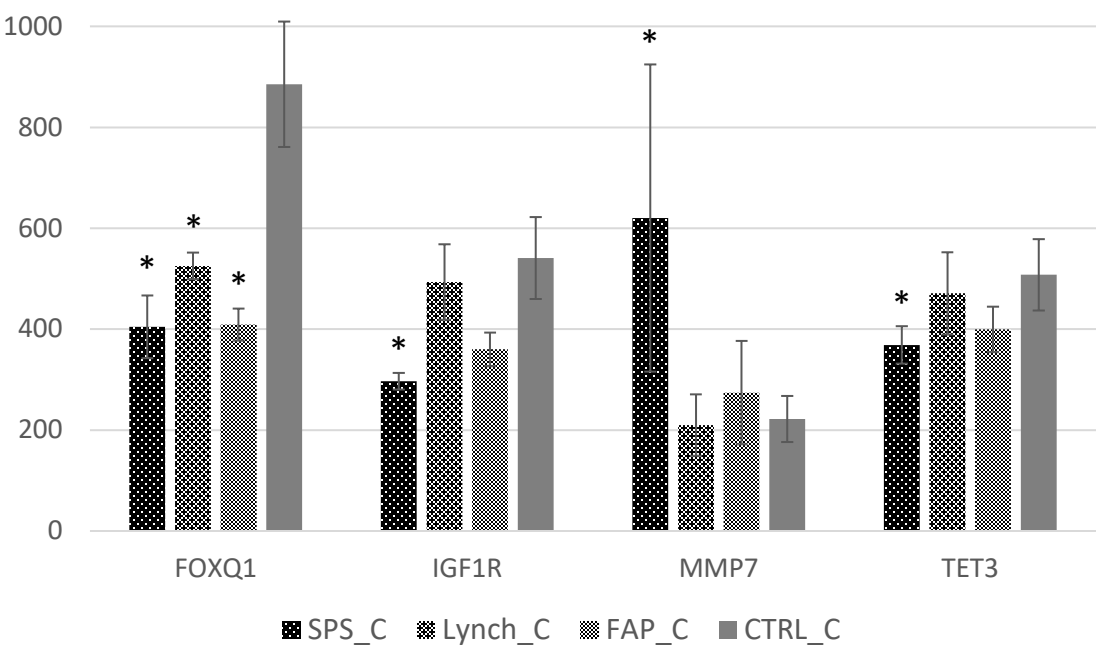

Supplement: Supplementary file 3 — Supplementary file3 (PDF 70 KB) [file 10689_2024_370_MOESM3_ESM.pdf]
