# Supplementary material for: Evaluation of EGFR and COX pathway inhibition in human colon organoids of serrated polyposis and other hereditary cancer syndromes
Source: Fam Cancer. 2024 Apr 12;23(4):479–89. doi: 10.1007/s10689-024-00370-7 (PMC11512843; doi:10.1007/s10689-024-00370-7)
Supplement: Supplementary file 8 — Supplementary file8 (DOCX 15 KB) [file 10689_2024_370_MOESM8_ESM.docx]

**Supplemental Legends and Methods**

**Figure S1.** Light microscopy 20X photomicrographs of uninvolved (non-polyp) colon organoids derived from syndromic patients. **Left**, Familial Adenomatous Polyposis (FAP; 359F-AC), **Right**, Lynch syndrome (LS; 352L-SC).

<https://figshare.com/s/5e4e3d1589de65e4feb6>

**Figure S2.** Antibody-stained human proximal polyp and uninvolved colon organoids, Day 5 post-passage. A-C) Anti-MUC2-antibody (red) and anti-PS6 antibody (green) -stained, bars denote 200 mm. A) SPS patient’s ascending normal colon from (uninvolved; culture 360S-AC); B) SPS patient’s ascending colon sessile serrated lesion, BRAF-mutant (polyp; culture 360S-AP); C) different SPS patient’s transverse colon sessile serrated lesion, BRAF-mutant (polyp; culture 357S-TP). D-F) Anti-MUC2-antibody (green) and anti-pAKT1 antibody (red) -stained, bars denote 200 mm. D) SPS patient’s ascending normal colon (uninvolved; culture 360S-AC); E) FAP patient’s cecal adenomatous polyp, BRAF-wildtype (polyp; culture 359F-CP); F) SPS patient’s ascending colon sessile serrated lesion, BRAF-mutant (polyp; culture 360S-AP).

<https://figshare.com/s/8f01488c48ae2198f59d>

**Figure S3.** RNA expression of *FOXQ1, IGF1R, MMP7* and *TET3* in control (C) organoids derived from uninvolved colon from SPS, FAP, Lynch and control patient cohorts. Bar graphs show the mean and standard error (n=4-6) of normalized read counts for each gene. Statistical significance determined by DESeq2, FDR < 0.05.

<https://figshare.com/s/3ffa7a40ee6d82744444>

**Table S1.** Custom NanoString mRNA gene panel including group category of gene marker. Group categories include COX, EGFR, PI3K and WNT signaling, colon cell type, field effect, housekeeping, non-coding and SSL gene markers.

<https://figshare.com/s/410ba4c58b7c7a94f9b7>

**Table S2.** Differentially expressed mRNAs by NanoString analysis in colon organoids based on BRAF status. Differential expression was determined using the DEseq2 R package. Values are expressed as log2 fold change and false discovery rate (FDR) after multiple testing correction (n=12 *BRAF WT* and n=3 *BRAF* mutant).

<https://figshare.com/s/e4ca7f96e40a685d8192>

**Table S3.** Differentially expressed microRNAs by NanoString analysis in colon organoids based on BRAF status. Differential expression was determined using the DEseq2 R package. Values are expressed as log2 fold change and false discovery rate (FDR) after multiple testing correction. (n=4 *BRAF WT* and n=3 *BRAF* mutant).

<https://figshare.com/s/a392409503270df4d11f>

Table S4. List of organoid cultures used for gene expression and morphological comparisons. For qualitative culture morphology comparisons, “+” and “++” signify present and severe, respectively. For gene expression comparisons, values indicate the number of samples per organoid culture used for statistical comparisons.

**Supplemental Methods.** Immunofluorescent staining of whole-mount organoids:

Organoids were grown to Day 5 post-passage in 24-well tissue culture plate wells in our routine growth media (no erlotinib nor sulindac), five 7ul MatriGel spots per well in 200ul media. Media was removed and 300ul 4% formaldehyde (Thermo Scientific 28908) in DPBS (Gibco 14190-144) was added for 20 min. Fixative was removed and each well was washed once with IF Buffer (0.2% Triton X-100 (Sigma-Aldrich T8787), 0.05% Tween-20 (Sigma-Aldrich P9416) in DPBS). IF Buffer was removed and 300ul permeabilization solution (0.5% Triton X-100 in DPBS) was added for 20 min. The well was washed once with 300ul IF Buffer, and 300ul blocking solution (1% BSA (Sigma Aldrich A7906) in IF Buffer) was added for 30 min. 200ul primary antibody in blocking solution (or only blocking solution, for secondary-only negative control wells (not shown)) was added, and plate was incubated 16-18 h at 4^o^C, in the dark in a humidified chamber. Primary solution was removed. Each well was washed 5 min 3X with 300ul IF Buffer, supplemented by gentle shaking in an orbital shaker. 250ul secondary antibody in blocking solution was added per well and plate was incubated in the dark 1 h at 22^o^C.  Each well was washed 5 min 3X with 300ul IF Buffer, again supplemented by gentle shaking. Fresh IF Buffer was added, and within 3 h staining was visualized and recorded with an EVOS FL Auto Imaging microscope system (Life Technologies AMAFD1000) set at monochrome-GFP for imaging the green Alexa Fluor 488 secondary antibody and monochrome-CY5 for imaging the red Alexa Fluor 647 secondary antibody. Imaging was performed at the Fluorescence Microscopy Core Facility, a part of the Health Sciences Cores at the University of Utah. All antibodies were obtained from Invitrogen/Thermo Fisher Scientific. Primary antibodies and their dilutions were: MUC2 monoclonal mouse, MA5-12345, 1:100; Phospho-S6 (Ser235, Ser236) monoclonal rabbit, MA5-15140, 1:100, Phospho-AKT1 (Ser473) monoclonal rabbit, 44-621G, 1:100. Secondary antibodies and their dilutions used in Figure 1 and/or Supplemental Figure 2 (A-C) were: Goat anti-mouse, Alexa Fluor 647 (red), A-21236, 1:1,000; Goat anti-rabbit, Alexa Fluor 488 (green), A11034, 1:1,000. Secondary antibodies and their dilutions used in Supplemental Figure 2 (D-F) were: Goat anti-mouse, Alexa Fluor 488 (green), R37120, 1:1,000; Goat anti-rabbit, Alexa Fluor 647 (red), A32733, 1:1,000.

<https://figshare.com/s/e4cf0874fbbe8798942c>
